# Molecular Typing of Tick-Borne Pathogens in Ixodids of Bosnia and Herzegovina

**DOI:** 10.3390/microorganisms13051054

**Published:** 2025-04-30

**Authors:** Ina Hoxha, Jovana Dervović, Margarida Ruivo, Michiel Wijnveld, Adelheid G. Obwaller, Bernhard Jäger, Martin Weiler, Julia Walochnik, Edwin Kniha, Amer Alić

**Affiliations:** 1 Institute of Specific Prophylaxis and Tropical Medicine, Center for Pathophysiology, Infectiology and Immunology, Medical University Vienna, 1090 Vienna, Austria; ina.hoxha@meduniwien.ac.at; 2 Department of Clinical Sciences of Veterinary Medicine, Faculty of Veterinary Medicine, University of Sarajevo, 71000 Sarajevo, Bosnia and Herzegovina; jovana.supic@vfs.unsa.ba (J.D.); amer.alic@vfs.unsa.ba (A.A.); 3 Institute for Hygiene and Applied Immunology, Center for Pathophysiology, Infectiology and Immunology, Medical University of Vienna, 1090 Vienna, Austria; margarida.ruivo@meduniwien.ac.at (M.R.); michiel.wijnveld@meduniwien.ac.at (M.W.); 4 Division of Science, Research and Development, Federal Ministry of Defence, 1090 Vienna, Austria; adelheid.obwaller@bmlv.gv.at; 5 CBRN Defence Centre, Austrian Armed Forces, 2100 Korneuburg, Austria; bernhard.jaeger@bmlv.gv.at (B.J.); martin.weiler@bmlv.gv.at (M.W.)

**Keywords:** *Anaplasma*, Balkan, barcoding, *Borrelia*, *Neoehrlichia mikurensis*, PCR, reverse line blotting, *Rickettsia*

## Abstract

Ticks are key vectors of zoonotic pathogens, and their expanding distribution in Europe heightens public health concerns. In Bosnia and Herzegovina, while tick distribution is well documented, molecular data on tick-borne pathogens remain limited. This study aimed to illustrate the presence and diversity of these pathogens, focusing on areas with high human activity. Ticks (n = 556) were collected in April 2022 from eight diverse locations, including urban parks, private properties, and rural sites. PCR-based screening was employed to detect Anaplasmataceae, *Borrelia*, *Francisella*, Piroplasmida, *Rickettsia*, and tick-borne encephalitis virus (TBEV), with subsequent sequencing to confirm results. Further characterization of *Borrelia burgdorferi* sensu lato was achieved via reverse line blotting (RLB) hybridization and sequencing. *Ixodes ricinus* was the most prevalent species, followed by *Dermacentor marginatus* and *D. reticulatus.* Our analysis revealed an overall infection rate of 22.1% in questing ticks, with *Rickettsia* spp. and *Borrelia* spp. predominating. Notably, seven *Borrelia* species were identified in *I. ricinus*, alongside *Anaplasma phagocytophilum*, *Rickettsia helvetica*, and *R. monacensis*, with co-infections mainly observed in peri-urban areas. This study provides the first molecular evidence of multiple tick-borne pathogens in the region, underscoring the need for further surveillance and risk assessment of tick-borne diseases in the region.

## 1. Introduction

Among all arthropod vectors, ixodid or hard ticks (Acari: Ixodida: Ixodidae) transmit the widest range of pathogens, including protozoa, bacteria, and viruses, posing significant medical and veterinary risks [1]. Hard ticks are widely present in Europe, and climate change is expanding their habitats and thus increasing the risk of regular contact to tick-borne pathogens (TBPs) for both animals and humans [2,3]. The prevalence of TBPs impacting human health has risen significantly over recent decades, reflecting a growing public health challenge [3].

*Ixodes ricinus* is a three-host tick and the most prevalent tick species throughout the western Palearctic, including Central and (south)eastern Europe. New locations are continuously recorded in Europe [4,5], and endemic areas have reported a notable increase in abundance [6]. Feeding patterns of this species are highly diverse, allowing ticks to feed on various animal species, from small mammals and birds to larger animals, which contributes to the transmission of various pathogens [7,8]. The recent description of *Ixodes inopinatus* as a distinct species from *I. ricinus* emphasizes the need for further investigations into its distribution and ecological role. Both species co-exist in overlapping geographical regions, including the Mediterranean basin; however, morphological and molecular identification is challenging [9,10].

The genus *Dermacentor* comprises 33 species; *Dermacentor marginatus* and *D. reticulatus* are the most abundant tick species in the Balkans. Both species quest by latching onto hosts from low vegetation [11]. *Dermacentor marginatus* commonly targets medium and large mammals including deer, whereas *D. reticulatus* targets livestock and wildlife, as it is found in open habitats [12,13].

*Ixodes ricinus* is a highly competent vector for a variety of pathogens, of which the spirochete bacteria *Borrelia* are the most prevalent. *Borrelia burgdorferi* s.l. display the most important and the most prevalent tick-borne pathogens in the Northern Hemisphere [14]. Species within the *B. burgdorferi* s.l. complex, including *B. burgdorferi* s.s., *Borrelia afzelii*, and *Borrelia garinii*, are associated with the progression of Lyme borreliosis (LB) due to their unique pathogenic profiles. *Borrelia afzelii* is commonly associated with persistent cutaneous symptoms, while *B. garinii* frequently leads to neurological complications [15,16]. The newly described species *B. spielmanii* has been identified in a few skin isolates from patients with erythema migrans [17], although its pathogenic potential remains to be fully investigated and confirmed.

Tick-borne encephalitis virus (TBEV) (*Flaviviridae*) presents a significant public health threat, and infections can cause neurological disorders, including meningitis, encephalitis, and meningoencephalitis [18]. *Ixodes ricinus* is the key vector for the transmission of the TBE virus across Europe and parts of Central Asia. Among ticks in the genus *Dermacentor*, the TBEV has also been detected in *D. marginatus* and *D. reticulatus*, the latter being a competent vector under experimental conditions [19], expanding its potential transmission range [20].

Other tick-borne bacteria of medical and particularly veterinary relevance are *Rickettsia*, *Anaplasma*, and *Ehrlichia*. The obligate intracellular bacteria of the genus *Rickettsia* are transmitted by *Ixodes* and *Dermacentor* ticks. The latter contributes to the spread of rickettsial pathogens that cause diseases such as spotted fever. Spotted fever group (SFG) *Rickettsia* spp., namely *R. slovaca* and *R. raoultii*, are the causative agents of tick-borne lymphadenopathy (TIBOLA) or *Dermacentor*-borne necrosis erythema and lymphadenopathy (DEBONEL) [21]. On the other hand, the role of *R. monacensis* in human disease remains unclear due to its limited pathogenicity despite its occasional detection in human cases [22,23], and the pathogenicity of *R. helvetica* has been suspected but never proven [24].

The genera *Anaplasma*, *Ehrlichia*, and two *Neoehrlichia* species comprise the intracellular bacteria of the Anaplasmataceae family that are transmitted by ticks to various mammalian hosts, including humans. Species such as *Anaplasma phagocytophilum*, *A. capra*, and *A. marginale* cause anaplasmosis with symptoms including fatigue, fever, and muscle aches; in severe cases, it may lead to complications like immune system dysregulation or reduced platelet levels (thrombocytopenia) [25].

Babesiosis, also known as piroplasmosis, is caused by intraerythrocytic protozoa of the genus *Babesia*. Symptoms often range from flu-like symptoms such as fever, chills, sweat, and fatigue, with severe cases potentially leading to hemolytic anemia due to red blood cell destruction. The risk of complications is higher in immune-compromised, splenectomised, or elderly individuals [26].

In Balkan countries, data on tick distribution are available, but tick-borne pathogens have been neglected for a long time. Tick surveillance in Bosnia and Herzegovina has demonstrated 19 endemic species [27], of which *I. ricinus* is the most prevalent species followed by *D. marginatus* [28]. A few studies, mostly including ticks collected from animals, revealed *B. burgdorferi* s.l. was predominantly detected in *I. ricinus* ticks, with detection rates ranging from 2 to 5% depending on the collection site and environmental factors [29]. Similarly, *Rickettsia* spp., including *R. slovaca* and *R. raoultii*, have been detected in *Dermacentor* and *Ixodes* ticks, with reported prevalence rates surpassing 10% [30]. Omeragić et al. [29] reported a minimal infection rate of 3.1% for *Babesia* and 8.8% for *A. phagocytophilum* in *I. ricinus* pools. These findings highlight the presence of pathogens in Bosnia and Herzegovina and a dynamic of infection that affects both animals and humans. Although surveys on tick distribution have been conducted, molecular data confirming pathogen presence by PCR and sequencing, particularly from individually analyzed specimens, are scarce but important to assess true prevalence rates. In the current study, we aimed to provide new insights into the prevalence of tick-borne pathogens and analyze sequence data in central and northeastern Bosnia and Herzegovina, particularly at locations with frequent human interaction such as recreational areas.

## 2. Materials and Methods

### 2.1. Tick Collection Sites

**Figure 1:**
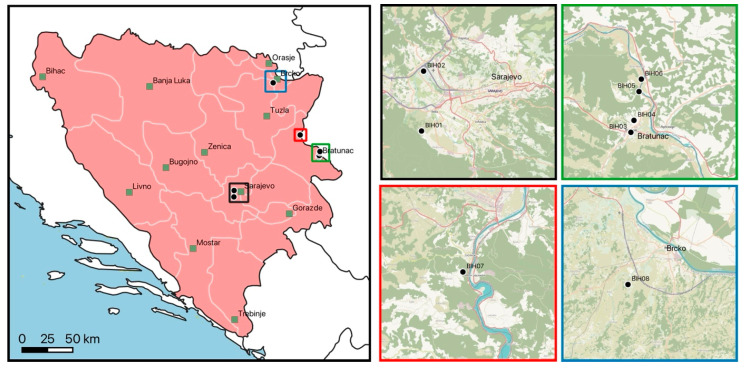
Collection sites in Bosnia and Herzegovina. Major cities are indicated as black-framed green squares. Colored squares in the main map are displayed as magnified areas on the right. Country borders, first-level administrative divisions, and sea data were included using Natural Earth (www.naturalearthdata.com, accessed on 28 November 2024).

The survey was conducted at eight different peri-urban and rural collection sites in central and northeastern Bosnia and Herzegovina. Collection sites were chosen based on potential suitability for questing ticks (e.g., uncut grass, meadow/forest intersection) and accessibility (public or private with access granted by owners) in April 2022 (Figure 1). All collection sites were sampled once. The attributes of collection sites are given in Table 1.

Questing ticks were collected using a 1 × 1 m white flannel flag, flagging along predefined transects (on average 10 × 50 m^2^) for an hour. At location BIH1, sheep close by were additionally sampled (with consent of the owners) by carefully removing engorged ticks from animal hosts using tweezers to ensure specimen integrity. Collected ticks were put in 2 mL tubes with screw caps, stored in dry ice before storage in −80 °C.

### 2.2. Nucleic Acid Extraction

For nucleic acid extraction, ticks were cut longitudinally into two halves. From one half, DNA was isolated using a DNeasy^®^ Blood and Tissue Kit (Qiagen, Hilden, Germany) by incubating in 180 µL ATL buffer and 20 µL proteinase K overnight following the manufacturer’s instructions.

For RNA isolation, halves of ticks were pooled (maximum of six individuals), sorted by location and sex. To each pool, 180 µL phosphate-buffered saline (PBS) was added and crushed with 3 mm stainless steel beads for 5 min at 6000× *g* with a Qiagen TissueLyser LT (Qiagen, Hilden, Germany). The homogenate was centrifuged at 18,000× *g* for 5 min, and the supernatant was transferred to a new tube. Consecutively, a Qiagen RNeasy Mini Kit (Qiagen, Hilden, Germany) was used, adding 560 µL of AVL-AVE Lysis buffer (containing RNA carrier) to the homogenate, followed by 350 µL of 70% ethanol, only mixing by pipetting. Subsequent steps were performed based on the manufacturer’s instructions, with a 50 µL final elution volume. The RNA eluate was stored at −20 °C until further use.

### 2.3. Morphological and Molecular Tick Identification

The morphological identification of ticks was performed under a stereomicroscope using the identification keys from Estrada-Peña et al. [31]. Due to morphological and genetic discrepancies between *I. ricinus* and *I. inopinatus*, we refer to *I. ricinus* sensu lato in this study.

The molecular identification of chosen ticks was based on the amplification of a 16S rRNA gene segment using the primer combination 16S+1 (5′-CTGCTCAATGATTTTTTAAATTGCTGTGG-3′) and 16S-1 (5′-CCGGTCTGAACTCAGATCAAGT-3′) by Black and Piesman [32]. The PCR conditions were 94 °C for 5 min for initial denaturation, followed by 38 cycles of 94 °C for 1 min, 52 °C for 1 min, and 72 °C for 1 min, with a final elongation at 72 °C for 10 min. For all PCR amplifications, the 2× EmeraldAmp^®^ GT PCR Master Mix (Takara Bio Europe, Paris, France) was used with 2 μL template DNA and sterile H_2_O, adding up to a final volume of 25 μL. Reactions were run on an Eppendorf Mastercycler (Eppendorf AG, Hamburg, Germany). Bands were analyzed with a Gel DocTM XR+ Imager (Bio-Rad Laboratories, Inc., Hercules, CA, USA), cut out and subsequently purified with the IllustraTM GFXTM PCR DNA and Gel Purification Kit (GE Healthcare, Buckinghamshire, UK); then, they were sent to Microsynth Austria GmbH for Sanger sequencing.

Sequences were obtained from both strands, aligned with Clustal X 2.1 [33], and a consensus sequence was generated in GenDoc 2.7.0 [34]. The obtained sequences were uploaded to GenBank and compared to available sequences in the GenBank database using the Basic Local Alignment Search Tool (BLAST) (https://blast.ncbi.nlm.nih.gov/Blast.cgi; accessed on 25 March 2025).

### 2.4. DNA-Based Pathogen Detection

Samples were screened for the presence of Anaplasmataceae, *Borrelia*, *Francisella*, Piroplasmida, and *Rickettsia* DNA. For the detection of pathogen DNA, published protocols were used (Table 2). Negative controls (H_2_O) and respective positive controls (DNA at our disposal of *Anaplasma marginale* originating from bovine blood, *Borrelia burgdorferi* s.s. B31 strain, *Babesia venatorum* originating from *I. ricinus*, *Rickettsia raoultii* Jongejan strain, and *Francisella tularensis* subsp. *holarctica*) were used in each PCR run.

### 2.5. RNA-Based Pathogen Detection

A reverse transcriptase (RT) qPCR with the “universal” flavivirus primer set PF1S (5′-TGYRTBTAYAACATGATGGG-3′) and PF2Rbis (5′-GTGTCCCADCCDGCDGTRTC-3′) and the Luna^®^ Universal One-Step RT-qPCR Kit (New England Biolabs, Ipswich, MA, USA) was used to detect TBEV RNA [46]. For all reactions, 2.5 µL of RNA template was used with the following PCR conditions: 55 °C for 10 min, followed by 45 cycles of 95 °C for 1 min, 95 °C for 10 s, and 50 °C for 1 min. Sterile H_2_O was used as a negative control, and RNA extracted from a live Yellow fever virus vaccine (Stamaril^®^, Sanofi, Paris, France) was used as a positive control.

### 2.6. Borrelia Burgdorferi Sensu Lato Species Discrimination by Reverse Line Blotting (RLB)

All samples positive for *B. burgdorferi* s.l. by PCR were subjected to PCR-RLB hybridization. A genus-specific RLB-PCR was carried out as reported previously [47,48], using the biotinylated primer pairs shown in Table 1. Each PCR reaction mix with a 25 μL total volume contained the following: 5 μL (5×) of Phire reaction buffer, 200 nmol/L of each dNTP (Solis BioDyne, Tartu, Estonia), 400 nmol/L of each primer per specific primer pair, 0.125 μL Phire Hot Start II DNA Polymerase (Thermo Scientific, Vienna, Austria), PCR-grade water (Sigma-Aldrich, Vienna, Austria), and 2.5 μL of template DNA [49]. The resulting amplicons were analyzed using RLB hybridization as described previously [50].

The further discrimination of *B. garinii* and *B. bavariensis* was achieved by amplification and sequencing of the housekeeping gene clpA [41]. A nested PCR was carried out using the primer pairs shown in Table 1. Each PCR reaction was prepared in a total of 25 μL volume containing the following: 5 μL (5×) of Phire reaction buffer, 200 nmol/L of each dNTP (Solis BioDyne, Tartu, Estonia), 400 nmol/L of each primer per specific primer pair (first reaction: clpAF1237 and clpAR2218 and second reaction: clpAF1255 and clpAR2104), 0.5 μL Phire Hot Start II DNA Polymerase (Thermo Scientific, Vienna, Austria), PCR-grade water (Sigma-Aldrich, Vienna, Austria), and lastly 2.5 μL of template DNA. Bands were analyzed with iBright CL750 Imaging System (Thermo Fisher Scientific, Vienna, Austria), cut out, and subsequently purified using QIAquick Gel Extraction kit (Qiagen, Hilden, Germany); then, they were sent to sequencing at Microsynth (Microsynth AG, Vienna, Austria).

### 2.7. Statistical Analysis

All data were analyzed using Microsoft Excel 16.82 for Mac and R 3.6.2 [51]. Due to the heterogeneity of prevalence rates between locations and the low number of *Dermacentor* spp., we refrained from further statistical analyses.

## 3. Results

### 3.1. Tick Collection

Overall, 556 ticks were collected, comprising three species from eight locations. Of these, 511 (91.9%) were *Ixodes ricinus*, 42 (7.6%) were *Dermacentor marginatus*, and 3 (0.5%) were *D. reticulatus*. Of all, 507 (91.2%) specimens were questing, 16 (2.9%) were unfed from a host, and 33 (5.9%) were engorged females (Table 3). Only *I. ricinus* and *D. marginatus* were collected from host animals, which were sheep exclusively at location BIH1 (Stojčevac public park).

*Ixodes ricinus* was collected at seven locations, except an open agricultural field (BIH2), and numbers varied considerably between locations. *Dermacentor marginatus* was sampled at three locations (engorged at BIH1 from sheep close to public park, questing at BIH2 open agricultural field, and BIH8 private property) and *D. reticulatus* collected at only one location (questing at BIH2 private property) (Table 4).

At location BIH1, *I. ricinus* and *D. marginatus* were both collected from sheep, and sympatric occurrence of questing *D. marginatus* and *D. reticulatus* was observed at location BIH2 (open agricultural field) (Table 4).

### 3.2. Tick Barcoding

Altogether, 16S rDNA barcoding of 70 specimens was performed. All barcodes allowed for identification to the species level. Haplotyping revealed no genetic pattern associated with geographical origin. Twenty-three *I. ricinus* sequences with a length of 406 to 408 base pairs (bp) resulted in 12 haplotypes, all showing 99.75% to 100% identity with various *I. ricinus* sequences originating, e.g., from Poland (MK671578), Slovakia (MN947216), and Portugal (KY039161). Thirty-four barcodes with a length of 414 to 416 bp were obtained from *D. marginatus*, divided into eight haplotypes, which showed 99.76% to 100% identity with sequences from China (OM368304), Turkey (MT229170), and Spain (MH645513). All three *D. reticulatus* sequences with a length of 403 bp displaying a single haplotype were 100% identical to reference sequences from Russia (OR936112) and Poland (MK671579) (Table 5).

### 3.3. DNA-Based Pathogen Screening

Altogether, 112 (112/507; 22.1%) questing ticks, nine (9/16; 56.3%) unfed ticks collected from hosts, and four (4/33; 12.1%) engorged ticks were positive for at least one pathogen. Double infections were detected in questing ticks (11/507; 2.2%) and unfed ticks from hosts (2/16; 12.5%), and triple infections (5/507; 1.0%) were only from questing *I. ricinus* ticks (Table 6).

Of 495 questing *Ixodes ricinus* specimens, 108 (21.1%) were infected with at least one pathogen, 13 (2.5%) were infected with two pathogens, and 5 (1.0%) with three pathogens (Table 6). Of all 16 engorged female *I. ricinus* collected from sheep, only one (6.3%) was infected with *R. monacensis*. The highest single infection rates were observed for *Rickettsia* (58/511; 11.4%) and *Borrelia* (39/511; 7.9%), followed by *Anaplasma* (15/511; 2.9%) and *Neoehrlichia mikurensis* (1/511; 0.2%) (Table 7).

For *Anaplasma*, only *A. phagocytophilum* was detected, with highest rates in female ticks (4.8%). *Borrelia* comprised *B. burgdorferi* s.l. (37/511; 7.2%), as well as *B. miyamotoi* (2/511; 0.4%). One sample (nymph) was positive for *Neoehrlichia mikurensis* (0.2%), and *Rickettsia* positive samples were split into *R. helvetica* (42/511; 8.2%) and *R. monacensis* (16/511; 3.3%) (Table 7).

Further discrimination of *B. burgdorferi* s.l. by RLB revealed six species, namely *B. afzelii* (10/511; 2.0%), *B. burgdorferi* s.s. (14/511; 2.7%), *B. garinii* (2/511; 0.4%), *B. lusitaniae* (18/511; 3.5%), *B. spielmanii* (2/511; 0.4%), and *B. valaisiana* (5/511; 1.0%) (Table 7).

The majority of double infections (7/11; 63.6%) detected in *I. ricinus* comprised two *B. burgdorferi* s.l. species, as well as three (3/11; 27.2%) *B. lusitaniae* + *R. helvetica* infections and one (1/11; 9.0%) *B. burgdorferi* s.s. + *N. mikurensis* infection (Table 7). Triple infections involved several *B. burgdorferi* s.l. species, *A. phagocytophilum*, and *R. monacensis*.

Neither *Babesia*/*Theileria* or *Francisella* DNA nor flavivirus RNA were detected in the samples.

In *D. marginatus*, five (5/16; 31.3%) male specimens collected from hosts were positive for *A. ovis* and three (3/7; 42.9%) questing females, three (3/17; 17.6%) unengorged females, and four (4/16; 25.0%) males from a host were positive for *R. raoultii*. One (1/7; 14.3%) questing female and two (2/16; 12.5%) males collected from a host were positive for *R. slovaca*. Double infections were only detected in males collected from a host, which comprised one (1/16; 6.3%) *A. ovis* + *R. raoultii* co-infection and one (1/16; 6.3%) *A. ovis* + *R. slovaca* infection.

Of the three *D. reticulatus* specimens collected questing, one (1/3; 33.3%) was infected with *R. raoultii*.

As a byproduct of the Anaplasmataceae PCR, *Wolbachia* spp. and *Candidatus* Midichloria mitochondrii DNA were detected and sequenced. In 25 of 507 (4.9%) questing *I. ricinus*, *Wolbachia* spp. DNA was detected, but only in ticks from location BIH4 (9/187; 4.8%) and BIH5 (16/132; 12.1%). The majority of *Wolbachia* spp. DNA was detected in nymphs (23/211; 10.9%) and only few females (1/152; 0.7%) and males (1/132; 0.8%). All *Wolbachia* sp. sequences showed highest similarity with *Wolbachia* subgroup A. Additionally, in six (6/152; 4.0%) questing female *I. ricinus*, *Candidatus* Midichloria mitochondrii DNA was detected.

### 3.4. Detected Pathogens by Location

**Figure 2:**
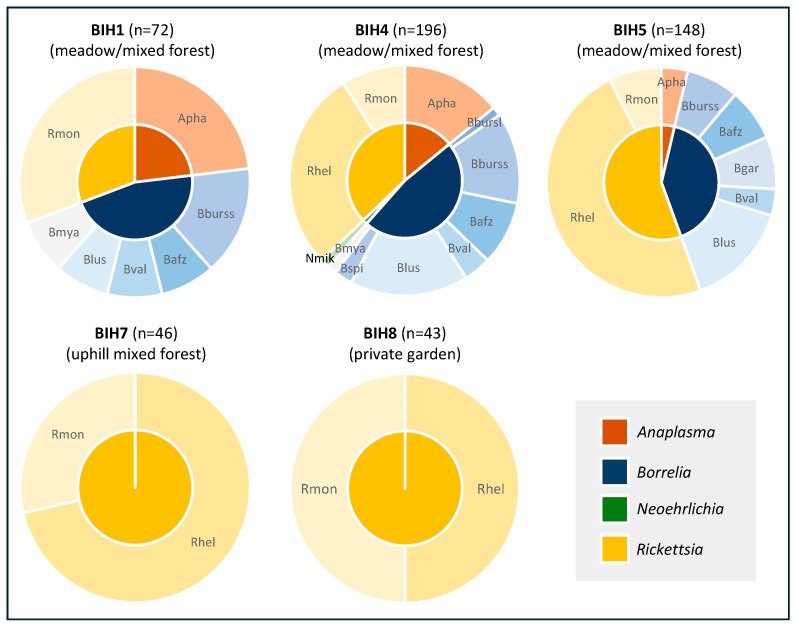
Pathogen diversity in questing *I. ricinus* by sampling location. Apha, *Anaplasma phagocytophilum*; Nmik, *Neoehrlichia mikurensis*; Bbursl, *Borrelia burgdorferi* s.l.; Bburss, *B. burgdorferi* s.s.; Bafz, *B. afzeli*; Bgar, *B. garinii*; Blus, *B. lusitaniae*; Bval, *B. valaisiana*; Bspi, *B. spielmanii*; Bmya, *B. myamotoi*; Rslo, *Rickettsia slovaca*; Rhel, *R. helvetica*; Rmon, *R. monacensis*.

While maintaining the same collection effort at all eight locations, the number of ticks and the number of detected pathogens varied considerably between locations. At two locations (BIH3 a private house with small garden and BIH6 along Drina riverbank), no pathogens were detected. At location BIH2 (around agricultural field), only *Dermacentor* spp. were detected, being infected with *R. raoultii* and *R. slovaca* (Supplementary Table S1). The highest diversity of pathogens in questing *I. ricinus* was detected at location BIH4 (10 pathogens), followed by location BIH5 (eight pathogens) and location BIH1 (seven pathogens), with all three peri-urban collection sites exhibiting the interface of meadows and mixed forests (Figure 2). Also, double and triple infections (BIH1 and BIH4) were only detected in *I. ricinus* from these three sites (Table S1).

### 3.5. Pathogen Typing

Sequences of pathogens were uploaded to GenBank and compared to available reference sequences. All 14 *A. phagocytophilum* sequences comprised a single haplotype, showing 100% identity with reference sequences originating from ticks (*I. ricinus*, MW922753; *I. scapularis*, HG916766; or *R. sanguineus* sensu lato, OR976127) and animals such as dogs (MK814412), horses (MK811374), or rats (OL690564). For *A. ovis*, the confirmatory PCR based on the major surface protein 4 (msp4) gene showed 100% identity with isolates from sheep in Pakistan (PQ616034) or China (MH908943) (Table 8).

For *Borrelia*, only *B. myamotoi* was subjected to sequence analysis, revealing 100% identity with reference sequences originating from *I. ricinus* in Czech Republic (KJ847049) and Poland (KF422749). Typing of *B. burgdorferi* s.l. species was accomplished by RLB only due to low specificity of the applied PCR.

A single sequence of *N. mikurensis* showed 99.26% identity to *Candidatus* Neoehrlichia sp. (OP269946, OP269947) and 98.9% identity to the sequence of the *Ca.* Neoehrlichia mikurensis reference genome (CP066557).

For *Rickettsia*, six *R. monacensis* sequences comprising two haplotypes were 99.71% to 100% identical to a strain from Poland (JQ796867) and the type strain (LN794217). Four *R. helvetica* sequences comprising two haplotypes showed 99.8% to 100% identity to reference sequences from Poland (JQ796866) and Austria (EU057990). Five *R. raoultii* sequences consisting of a single haplotype showed 100% identity to the “Khabarovsk” strain (CP010969), and three *R. slovaca* sequences (single haplotype) were 100% identical to strains from Pakistan (MN581971) and Portugal (AY125009) (Table 8).

## 4. Discussion

Our study provides a snapshot and a valuable cross-section through tick-borne pathogens in local tick populations from central and northeastern Bosnia and Herzegovina, thereby demonstrating a high diversity of pathogens. *Ixodes ricinus*, the predominant tick species in the area, showed an infection rate of more than 20% with at least one pathogen, including co-infections (2.5%) and triple infections (1.0%). We provide the first molecular data on *Borrelia* genotyping in the region, identifying six distinct *B. burgdorferi* sensu lato genospecies in addition to *B. myamotoi*.

### 4.1. Pathogens in Questing Ticks

Previous studies in Bosnia and Herzegovina have confirmed the presence of *B. burgdorferi* s.l.; however, the identification of specific genospecies has been limited. Omeragić et al. [29] conducted molecular screening of tick-borne pathogens in *I. ricinus* ticks, detecting *B. burgdorferi* s.l. in 3.4% of specimens exclusively in those collected from domestic animals. On the contrary, our study is the first to report the presence of six distinct species within the *B. burgdorferi* complex in this region, namely *B. afzelii*, *B. garinii*, *B. burgdorferi* s.s., *B. lusitaniae*, *B. spielmanii*, *B. valaisiana*, and additionally *B. miyamotoi*, a relapsing fever spirochete considered an emerging human pathogen [52]. Among the genospecies that infect humans, *B. burgdorferi* s.s., *B. afzelii*, and *B. garinii* are the primary agents of Lyme borreliosis in Europe [53,54]. Recently, Lasić et al. [55] detected *B. burgdorferi* s.l. in *I. ricinus* ticks collected from patients in Sarajevo, the capital of Bosnia and Herzegovina, and recent data from the Institute of Public Health [56] indicate a rising trend in reported Lyme borreliosis cases.

In Bosnia and Herzegovina, previous studies have identified the presence of *Rickettsia* species in ticks. For instance, Omeragić et al. [29] detected *Rickettsia* spp. in ticks collected from cats, sheep, goats, and dogs, while all ticks collected from vegetation tested negative. In contrast, our study found the highest *Rickettsia* spp. positivity rates in ticks sampled by flagging, with *R. helvetica* (8.2%) as the most prevalent, followed by *R. monacensis* (3.3%). This is in line with Hodžić et al. [57], who reported *R. helvetica* (5.7%) and *R. monacensis* (1.1%) in questing *I. ricinus* in the region. The molecular detection of *Rickettsia* spp. was reported in patients with a history of tick bites who sought medical care, confirming the presence of *R. helvetica*, *R. monacensis*, and *R. felis*, highlighting their potential pathogenicity in humans [58]. Boretti et al. [59] assessed the public health significance of *R. helvetica*, detecting the pathogen in dogs, foxes, humans, and *Ixodes* ticks. *Rickettsia helvetica* infections in humans manifest as a non-specific febrile illness and in more severe cases, *R. helvetica*-associated meningitis has been reported [60,61]. On the other hand, *R. monacensis* infections can present with fever, rash, or headache, though their full clinical spectrum remains incompletely understood [62].

DNA of *A. phagocytophilum* was detected in 3% of questing *I. ricinus*, contrary to a recent study by Omeragić et al. [29], who reported absence in pools of questing adult *I. ricinus*. However, the presence of *A. phagocytophilum* DNA was recently confirmed in various animal hosts, as well as in ticks collected from dogs across all regions of BIH, with infections ranging from 0.9 to 23.8% [29]. As an emerging tick-borne pathogen, *A. phagocytophilum* represents a significant veterinary and public health concern [63]. The low *A. phagocytophilum* infection rates observed in this study are in line with previous findings in Bosnia and Herzegovina [57] and more broadly across Eastern Europe. Although there are no officially reported human cases of *A. phagocytophilum* infections, the presence of *Anaplasma* spp. has been confirmed in blood samples collected from stray dogs in Bosnia and Herzegovina [64], which highlights the need for further surveillance and awareness of this emerging pathogen, particularly given its zoonotic potential [63] and the role of domestic animals as potential reservoirs. *Neoehrlichia mikurensis* was detected in only a single *I. ricinus* tick, highlighting its rare occurrence in the sampled tick population. This emerging tick-borne pathogen is of clinical relevance, particularly in immunocompromised individuals [65].

We report the detection of *Wolbachia* spp. and *Candidatus* Midichloria mitochondrii as a rather incidental finding while screening for TBPs of the genera *Anaplasma* and *Ehrlichia*, suggesting that the true prevalence of these symbionts might be higher if specific PCR assays were employed. *Wolbachia* subgroup A in ticks is typically associated with insect endoparasitoids like *Ixodiphagus hookeri*, which parasitize ticks, leading to incidental detection in tick samples [66]. *Midichloria* species are known endosymbionts of ticks, residing in ovarian tissues and occasionally in salivary glands, with potential implications for tick biology and pathogen transmission [67].

### 4.2. Co-Infections

Double and triple co-infections with *Borrelia* spp. or other tick-borne pathogens are more common in *I. ricinus* due to its diverse host range, and they may exacerbate disease severity in infected individuals [68,69,70]. In the present study, *Borrelia*-infected ticks were exclusively collected from peri-urban locations with frequent human presence, aligning with the findings on *I. ricinus* ticks collected from peri-urban locations in Kosovo [71]. The proximity to forested areas, the presence of wildlife species, and the presence of stray dogs likely contribute to the maintenance and transmission of *Borrelia* spp. and other tick-borne pathogens [72].

### 4.3. Pathogen Absence

Interestingly, no *Babesia* spp. DNA was detected in the analyzed tick samples, aligning with previous studies that reported low prevalence or sporadic detection in the region [73]. While *I. ricinus* is a known vector of *Babesia* spp., with human infections reported in Europe, its circulation in Bosnia and Herzegovina remains poorly characterized. Similarly, no evidence of tick-borne encephalitis virus (TBEV) was found, which may be attributed not only to inactive TBEV foci and a lower risk of transmission in the sampled areas but also to the relatively low number of ticks examined, given that TBEV infection rates in ticks are typically < 1:1000. However, considering the particular focal distribution of the TBEV and the endemic presence in neighboring countries, continued surveillance is crucial for assessing potential spillover risks [74,75].

### 4.4. Sympatric Occurrence of Dermacentor *spp.*

We observed sympatric occurrences of both *D. marginatus* and *D. reticulatus* species questing at the same locations, a phenomenon that has been documented in other regions. For instance, Drehmann et al. [76] reported overlapping distributions of these species across Germany, suggesting potential ecological interactions and shared habitats. While previous research confirms the presence of both *Dermacentor* species, Omeragić et al. [28] also noted that the previously registered abundance of *D. marginatus* had nearly doubled. In our study, *A. ovis* was detected in 31.3% of male *D. marginatus* ticks from hosts, while *R. raoultii* and *R. slovaca* were identified in both questing and host-collected ticks.

### 4.5. Differences in Pathogen Diversity

The absence or low diversity of tick-borne pathogens in rural locations may be attributed to limited sample sizes, reducing the likelihood of encountering infected ticks. However, pathogen prevalence is not solely dependent on sample size; host-related factors, including the availability, density, and reservoir competence of the main tick hosts also play a crucial role in pathogen circulation [77,78]. For instance, despite similar trapping effort, tick density and pathogen prevalence varied between the peri-urban and rural settings, as the difference in habitat supports distinct wildlife reservoirs and host communities, which affect tick feeding and infection cycles [72,79]. Low pathogen detection in certain locations may be attributed to the absence of key reservoir hosts, reducing pathogen circulation in local tick populations [6]. Areas with fragmented landscapes and lower host densities have been linked to decreased infection rates in ticks [80]. Additionally, climatic factors such as temperature, precipitation, and humidity play a crucial role in tick survival and pathogen transmission. For example, one site with few collected specimens and no pathogen detection in this study was located along the Drina River, where periodic flooding may disrupt tick activity and reduce pathogen persistence in the environment [81,82].

## 5. Conclusions

Despite sampling only once per location within a single year and the collection sites being heterogeneous, potentially influencing pathogen detection rates, our findings underscore the veterinary and medical significance of tick-borne pathogens in Bosnia and Herzegovina, particularly *Borrelia* species (including cases of co-infection), *A. phagocytophilum*, *N. mikurensis*, and *Rickettsia* spp., all of which were detected in our study. Continuous surveillance is essential, especially in recreational areas, where human–tick encounters are frequent, to monitor the dynamics of TBP circulation. Future research should aim for longitudinal sampling over multiple seasons across diverse habitats to better understand the dynamics of tick-borne diseases and their implications for public health and veterinary medicine.

## Figures and Tables

**Table 1:** Attributes of tick collection sites.

Location	Site	Type	Attributes	Collection Method
BIH1	public	peri-urban	large public recreational park with large meadows and forest close by, sheep close by	flagging, from host
BIH2	agricultural	rural	surroundings of agricultural area with high grass and bushes	flagging
BIH3	private	peri-urban	private property with uncut grass in small garden	flagging
BIH4	private	peri-urban	private property with large meadows and forest close by	flagging
BIH5	private	peri-urban	private property with large meadows and forest close by	flagging
BIH6	public	rural	riverbank along Drina, regular floodings	flagging
BIH7	public	rural	uphill forest around Zvornik castle	flagging
BIH8	private	peri-urban	private property with large meadows and forest close by	flagging

**Table 2:** PCR protocols for DNA-based pathogen detection.

Pathogen, Target Gene (Length)	Primer Sequence (5′-3′)	PCR Protocol	References
Anaplasmataceae 16S rRNA gene (345 bp)	EHR16SD-for: GGTACCYACAGAAGAAGTCC EHR16SR-rev: TAGCACTCATCGTTTACAGC	95 °C/2 min; 35×: 94 °C/1 min, 54 °C/30 s, 72 °C/30 s; 72 °C 5 min	[35,36]
*Anaplasma* spp. typing *A. capra* groEL gene (874 bp)	groEL for: TGAAGAGCATCAAACCCGAAG groEL rev: CTGCTCGATGCTATCGG	94 °C/5 min; 35×: 94 °C/30 s, 63 °C/30 s, 72 °C/1 min; 72 °C 10 min	[37]
*A. bovis* groEL gene (529 bp)	groEL for: GTGGGATGTACTGCTGACC groEL rev: ATGGGGAGATATCCGCGA	94 °C/5 min; 35×: 94 °C/30 s, 63 °C/30 s, 72 °C/1 min; 72 °C 10 min	[38]
*A. ovis* msp4 gene (347 bp)	msp4 for: TGAAGGGAGCGGGTCATGGG msp4 rev: GAGTAATTGCAGCCAGGCACTCT	94 °C/5 min; 35×: 94 °C/30 s, 63 °C/30 s, 72 °C/1 min; 72 °C 10 min	[39]
*A. phagocytophilum* 16S rRNA gene (172 bp)	16S-for: AGTGCTGAATGTGGGGATAATTTATCTCCGTG 16S-rev: CTAATCTCCATGTCAAGAGTGGTAAGGTTT	94 °C/5 min; 35×: 94 °C/30 s, 63 °C/30 s, 72 °C/1 min; 72 °C 10 min	[38]
*Borrelia* spp. 16S rRNA gene (674 bp)	BORR_ALLG_for: ACGCTGGCAGTGCGTCTT BORR_ALLG_rev: CTGATATCAACAGATTCC	94 °C/5 min; 40×: 94 °C/1.5 min, 63 °C/2 min, 72 °C/2 min; 72 °C 10 min	[40]
*B. garinii/* *B. bavariensis* typing * clpA gene (849 bp) clpA gene (706 bp)	clpAF1237: AAAGATAGATTTCTTCCAGAC clpAR2218: GAATTTCATCTATTAAAAGCTTTC clpAF1255: GACAAAGCTTTTGATATTTTAG clpAR2104: CAAAAAAAACATCAAATTTTCTATCTC	98 °C/1 min; 10×: 98 °C/5 s, (60–1 °C/cycle)/5 s, 72 °C/15 s; 40×: 98 °C/5 s, 50 °C/5 s, 72 °C/15 s; 72 °C 1 min 98 °C/1 min; 10×: 98 °C/5 s, (60 –1 °C/cycle)/5 s, 72 °C/15 s; 45×: 98 °C/5 s, 50 °C/5 s, 72 °C/15 s; 72 °C 1 min	[41]
*Francisella* 16S rRNA gene (400 bp)	TUL4-435: GCTGTATCATCATTTAATAAACTGCTG TUL4-863: TTGGGAAGCTTGTATCATGGCACT	94 °C/5 min; 40×: 94 °C/1 min, 54 °C/1 min, 72 °C/1 min; 72 °C/10 min	[42]
Piroplasmida * 18S rRNA gene (700 bp) 18S rRNA gene (561 bp)	BTH-1F: CCTGAGAAACGGCTACCACATCT BTH-1R: TTGCGACCATACTCCCCCCA GF2: GYYTTGTAATTGGAATGATGG GR2: CCAAAGACTTTGATTTCTCTC	94 °C/2 min; 40×: 95 °C/30 s, 68 °C/1 min, 2 °C/1 min; 72 °C 10 min 94 °C/2 min; 40×: 95 °C/30 s, 60 °C/1 min, 72 °C/1 min; 72 °C 10 min	[43,44]
*Rickettsia* 23S/5S rRNA gene (350–550 bp)	ITS_F: GATAGGTCGGGTGTGGAAG IST_R: TCGGGATGGGATCGTGTG	96 °C/4 min; 35×: 94 °C/1 min, 52 °C/1 min, 72 °C/2 min; 72 °C 3 min	[45]

* Nested PCR.

**Table 3:** Number of collected ticks by species, sex, and developmental stage.

	Nymph		Female			Male	
	Questing	Host	Questing	Host ^a^	Engorged ^b^	Questing	Host
*I. ricinus*	211	-	152	-	16	132	-
*D. marginatus*	-	-	7	-	17	2	16
*D. reticulatus*	-	-	3	-	-	-	-
Total	211	0	162	0	33	134	16

^a^ Unfed, collected from host. ^b^ Attached to host and fed.

**Table 4:** Sampled tick species by location.

	BIH1	BIH2	BIH3	BIH4	BIH5	BIH6	BIH7	BIH8	Total
*I. ricinus*	72	-	5	196	148	3	46	41	511
*D. marginatus*	34	6	-	-	-	-	-	2	42
*D. reticulatus*	-	3	-	-	-	-	-	-	3
Total	106	9	5	196	148	3	46	43	556

**Table 5:** Generated barcodes, haplotypes, and accession numbers of ticks from BIH.

Species	Barcodes	Haplotypes	Accession Numbers	BLAST Identity
*I. ricinus*	23	12	PV203446 to PV203468	99.75% (MK671578) to 100% (KY039161)
*D. marginatus*	34	8	PV203469 to PV203502	99.52% (OM368304) to 100% (MT229170)
*D. reticulatus*	3	1	PV203503 to PV203505	100% (OR936112)

**Table 6:** Detected pathogens by feeding status.

Status	Negative	Positive	Single	Double	Triple
questing (507)	395 (77.9%)	112 (22.1%)	105 (20.6%)	11 (2.2%)	5 (1.0%)
unfed from host (16)	7 (43.8%)	9 (56.3%)	7 (43.8%)	2 (12.5%)	-
engorged (33)	33 (87.9%)	4 (12.1%)	4 (12.1%)	-	-

**Table 7:** Single pathogens and co-infections detected in questing *I. ricinus* ticks.

Pathogen	Nymphs (n = 211) ^a^	Female (n = 152) ^a^	Male (n = 132) ^a^	Total (n = 495) ^b^
Single infection				
*A. phagocytophilum*	3 (1.4%)	8 (5.3%)	4 (3.0%)	15 (3.0%)
*B. burgdorferi* s.l. ^c^	1 (0.5%)	-	-	1 (0.2%)
*B. afzelii*	2 (1.0%)	5 (3.3%)	3 (2.3%)	10 (2.0%)
*B. burgdorferi* s.s.	8 (3.8%)	4 (2.6%)	2 (1.5%)	14 (2.8%)
*B. garinii*	1 (0.5%)	-	1 (0.8%)	2 (0.4%)
*B. lusitaniae*	2 (1.0%)	9 (5.9%)	7 (5.3%)	18 (3.6%)
*B. spielmanii*	-	-	2 (1.5%)	2 (0.4%)
*B. valaisiana*	2 (1.0%)	1 (0.7%)	2 (1.5%)	5 (1.0%)
*B. miyamotoi*	2 (1.0%)	-	-	2 (0.4%)
*N. mikurensis*	1 (0.5%)	-	-	1 (0.2%)
*R. helvetica*	11 (5.2%)	17 (11.2%)	14 (10.6%)	42 (8.5%)
*R. monacensis*	7 (3.3%)	3 (2.0%)	6 (4.6%)	16 (3.2%)
Double infection				
*B. burgdorferi* s.s. + *B. valaisiana*	1 (0.5%)	-	-	1 (0.2%)
*B. burgdorferi* s.s. + *B. afzelii*	-	1 (0.7%)	-	1 (0.2%)
*B. burgdorferi* s.s. + *B. lusitaniae*	-	1 (0.7%)	1 (0.8%)	2 (0.4%)
*B. afzelii* + *B. lusitaniae*	-	2 (1.3%)	-	2 (0.4%)
*B. lusitaniae* + *B. valaisiana*	-	-	1 (0.8%)	1 (0.2%)
*B. burgdorferi* s.s. + *N. mikurensis*	1 (0.5%)	-	-	1 (0.2%)
*B. lusitaniae* + *R. helvetica*	-	1 (0.7%)	2 (1.5%)	3 (0.6%)
Triple infection				
*B. burgdorferi* s.s. + *B. afzeli* + *B. lusitaniae*	2 (1.0%)	-	-	2 (0.4%)
*B. afzeli* + *B. valaisiana* + *B. spielmanii*	-	-	1 (0.8%)	1 (0.2%)
*R. monacensis* + *B. burgdorferi* s.s. + *B. lusitaniae*	-	-	1 (0.8%)	1 (0.2%)
*A. phagocytophilum* + *B. burgdorferi* s.s. + *B. lusitaniae*	-	1 (0.7%)	-	1 (0.2%)

^a^ No. and % of infected stage. ^b^ Total no. and % infected. ^c^ Not further discriminated by RLB.

**Table 8:** Generated sequences, haplotypes, and accession numbers of detected pathogens.

Pathogen	Sequences	Haplotypes	Accession Numbers	BLAST Identity
*A. phagocythophilum*	14	1	PV203568 to PV203581	100% (MW922753, HG916766, OL690564)
*A. ovis* ^a^	5	1	PV203582 to PV203586	100% (PQ616034, MH908943)
*B. myamotoi*	1	1	PV203587	100% (KJ847049, KF422749).
*N. mikurensis*	1	1	PV203588	100% (OP269946, OP269947)
*R. monacensis*	6	2	PV231331 to PV231336	99.71% to 100% (JQ796867, LN794217)
*R. helvetica*	4	2	PV231337 to PV231340	99.8% to 100% (JQ796866, EU057990)
*R. raoultii*	5	1	PV231341 to PV231345	100% (CP010969)
*R. slovaca*	3	1	PV231346 to PV231348	100% (MN581971, AY125009)

^a^ Sequences confirmed by PCR based on major surface protein 4 (msp4) gene.

## Data Availability

All data generated and analyzed during this study are included in the article and Supplementary Materials.

## Supplementary Materials

The following supporting information can be downloaded at: https://www.mdpi.com/article/10.3390/microorganisms13051054/s1, Table S1: Detected pathogens by sampling location.
