# Supplementary material for: Molecular Typing of Tick-Borne Pathogens in Ixodids of Bosnia and Herzegovina
Source: Microorganisms. 2025 Apr 30;13(5):1054. doi: 10.3390/microorganisms13051054 (PMC12114401; doi:10.3390/microorganisms13051054)
Supplement: Supplementary file 1 [file microorganisms-13-01054-s001.zip › microorganisms-3542014-supplementary.pdf]

Supplementary Table S1. Detected pathogens by sampling location.

	BIH1 (n=106)	BIH2 (n=9)[a]	BIH3 (n=5)	BIH4 (n=196)	BIH5 (n=148)	BIH6 (n=3)	BIH7 (n=46)	BIH8 (n=43)
**n infections**								
**single**	18	4	-	50	21	-	7	5
**double**	3	-	-	8	4	-	-	-
**triple**	1	-	-	4	-	-	-	-
Anaplasma								
A. phagocytophilum	3	-	-	11	1	-	-	-
A. ovis	5	-	-	-	-	-	-	-
Borrelia								
B. burgorferi s.l.	-	-	-	1	-	-	-	-
B. burgdorferi s.s.	2	-	-	10	2	-	-	-
B. afzeli	1	-	-	7	2	-	-	-
Bbav/Bgar	-	-	-	-	2	-	-	-
B. valaisiana	1	-	-	3	1	-	-	-
B. lusitaniae	1	-	-	13	4	-	-	-
B. spielmanni	-	-	-	2	-	-	-	-
B. myamotoi	1	-	-	1	-	-	-	-
Neoehrlichia								
C. N. mikurensis	-	-	-	1	-	-	-	-
Rickettsia								
R. helvetica	-	-	-	22	13	-	5	2
R. monacensis	4	-	-	7	2	-	2	2
R. raoultii	7	3	-	-	-	-	-	1
R. slovaca	2	1	-	-	-	-	-	-

[a]only *Dermacentor* collected
